# The *Dynamo* package for tomography and subtomogram averaging: components for *MATLAB*, GPU computing and EC2 Amazon Web Services

**DOI:** 10.1107/S2059798317003369

**Published:** 2017-04-20

**Authors:** Daniel Castaño-Díez

**Affiliations:** a BioEM Lab at C-CINA, Biozentrum, University of Basel, Matenstrasse 26, CH-4058 Basel, Switzerland

**Keywords:** cryo-electron tomography, subtomogram averaging, GPU computing, cloud computing, Amazon EC2

## Abstract

*Dynamo* is a package for the processing of tomographic data. As a tool for subtomogram averaging, it includes different alignment and classification strategies. Furthermore, its data-management module allows experiments to be organized in groups of tomograms, while offering specialized three-dimensional tomographic browsers that facilitate visualization, location of regions of interest, modelling and particle extraction in complex geometries. Here, a technical description of the package is presented, focusing on its diverse strategies for optimizing computing performance. *Dynamo* is built upon mbtools (middle layer toolbox), a general-purpose *MATLAB* library for object-oriented scientific programming specifically developed to underpin *Dynamo* but usable as an independent tool. Its structure intertwines a flexible *MATLAB* codebase with precompiled C++ functions that carry the burden of numerically intensive operations. The package can be delivered as a precompiled standalone ready for execution without a *MATLAB* license. Multicore parallelization on a single node is directly inherited from the high-level parallelization engine provided for *MATLAB*, automatically imparting a balanced workload among the threads in computationally intense tasks such as alignment and classification, but also in logistic-oriented tasks such as tomogram binning and particle extraction. *Dynamo* supports the use of graphical processing units (GPUs), yielding considerable speedup factors both for native *Dynamo* procedures (such as the numerically intensive subtomogram alignment) and procedures defined by the user through its *MATLAB*-based GPU library for three-dimensional operations. Cloud-based virtual computing environments supplied with a pre-installed version of *Dynamo* can be publicly accessed through the Amazon Elastic Compute Cloud (EC2), enabling users to rent GPU computing time on a pay-as-you-go basis, thus avoiding upfront investments in hardware and longterm software maintenance.

## Abstract 2

A technical description of the *Dynamo* software package for subtomogram averaging is provided. Details are given on advanced *MATLAB* libraries, parallelization strategies, the use of GPUs and accessibility though the Amazon cloud computing services.

## 1. Introduction   

The cryo-electron tomography technique allows the imaging of cellular structures in a close-to-native context (Lučić *et al.*, 2005 ▸, 2013 ▸), representing them as three-dimensional models called tomograms. Copies of a given macromolecular structure can be located and averaged together by the technique known as subtomogram averaging, which aligns the noisy copies with their common signal, possibly classifying different conformational states (Briggs, 2013 ▸). The scope of the attainable level of detail has dramatically increased since the introduction of direct detector cameras, with several reports of resolutions below one nanometre (Schur *et al.*, 2015 ▸; Khoshouei *et al.*, 2016 ▸; Pfeffer *et al.*, 2015 ▸) or even close to the atomic level (Schur *et al.*, 2016 ▸).

The general workflow of subtomogram averaging and the algorithms behind it have been explained on a general level in several reviews (Wan & Briggs, 2016 ▸), while individual teams of developers have described the particularities of their different packages: the *IMOD*-integrated *PEET* (Heumann *et al.*, 2011 ▸), *Jsubtomo* (Huiskonen *et al.*, 2014 ▸), *EMAN*2 (Galaz-Montoya *et al.*, 2015 ▸), *XMIPP* (Scheres *et al.*, 2009 ▸) and *RELION* (Bharat *et al.*, 2015 ▸). The functionalities of *Dynamo* have also been described in detail in Castaño-Díez *et al.* (2012 ▸), which addressed the alignment and classification methods, and in Castaño-Díez *et al.* (2017 ▸), which reported the data-management system for visualization and annotation of sets of tomograms.

After a brief summary of the main functionalities currently included in *Dynamo*, this report focuses on more technical details of the software implementation, describing the architecture of the package and its implementation strategies for different scenarios: single and multinode parallelization, GPU computation and adaption to cloud computing through Amazon Web Services (AWS).

## 2. Functionalities   

### 2.1. Subtomogram averaging   

We introduce here some notation to be used throughout this manuscript. Within *Dynamo*, a subtomogram-averaging procedure consists of the analysis of *N* particles (each with a side length of *L* voxels) using *K* templates. *Alignment of a particle* against a template consists of performing a loop of *R* rotations of the template. At each step of the loop, the rotated template and particle are both bandpassed to the same resolution, and the rotated template is filtered with the particle’s missing wedge to keep only the Fourier coefficients present in the data. This operation is followed by computation of the locally normalized cross-correlation (Roseman, 2003 ▸) of all allowed translations of the rotated template against the data particle. The result is a single set of alignment parameters (three components of a shift vector and three Euler angles) and a cross-correlation coefficient, selected as the rotation and shift that yield the maximum cross-correlation over all tested rotations and translations.

In the *averaging* step, the particles with the best correlation may be selected for averaging. The resulting averaged particle is used as the alignment reference in the next iteration.

In multireference alignment (MRA), the alignment process runs independently on different references. On each reference *k*, the averaging step considers only those particles for which the highest cross-correlation was attained when aligned against that reference.

### 2.2. Implementation   

The core concept when using *Dynamo* for subtomogram averaging is the *project*. A project links and structures as a single entity all of the elements needed to effectively run the iterative procedure for alignment:

(i) the input files: the location of the data (with possible initial orientations), descriptions of the missing wedges, templates and masks;
(ii) the numerical settings, mainly the determination of scanned angular sets, policies for threshold selection, allowed shifts and possible binning;
(iii) the computation settings, establishing the number of CPU cores and/or GPU units to use. If a remote cluster is used, the project will guide the user towards the creation of a *configuration file* that will allow *Dynamo* to handle the communication with the queuing system.

**Figure 1:**
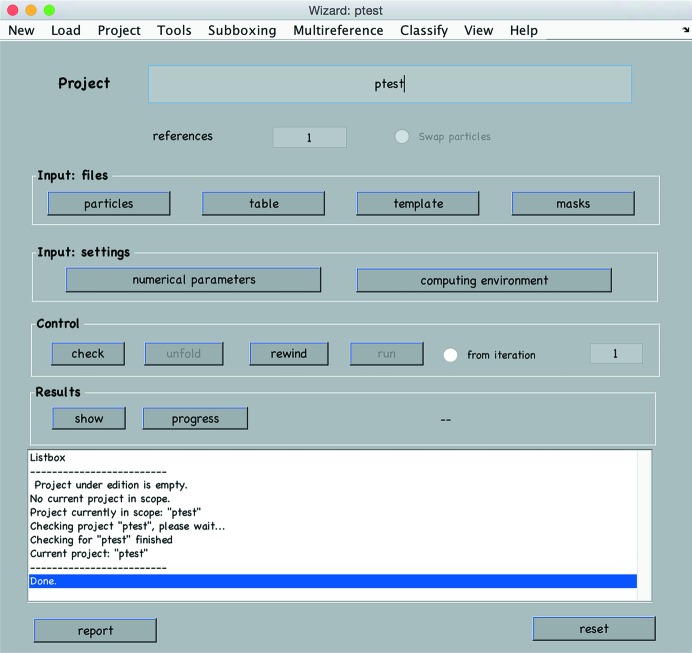
User interface for subtomogram-averaging projects.

*Dynamo* provides several tools for project manipulation. Along with flexible command-line options for more advanced users, the GUI dcp provides a graphical interface for controlling the creation of a project from scratch, executing it and accessing the results (Fig. 1 ▸).

### 2.3. Data management   

The second main functionality of *Dynamo* concerns the logistics of the steps leading to subtomogram averaging: the organization and annotation of sets of tomograms from which the subtomogram particles will be cropped into a data folder. Initial orientations of the particles will be expressed in the table accompanying the data folder.

This functionality area is handled by the *Catalogue* module. A *catalogue* is a set of tomograms indexed by the *catalogue manager*. Users can create a catalogue that manages a set of tomograms by passing a text file containing the names of the tomograms to the catalogue manager, dcm -create myCatalogue -fromStar myTomograms.star, or equivalently through the corresponding GUI.

After creation of the catalogue, the tomograms can be accessed independently (for visualization and annotation) and jointly (for the extraction of subtomograms towards the creation of a subtomogram-averaging project).

### 2.4. Visualization   

*Dynamo* includes different task-tailored tools for the visualization of tomograms. Navigation offline inside huge tomograms can be performed smoothly by *dtmshow*, which loads into memory only the fraction of tomogram required for a given depicted scene. *dpreview* allows fragments of interest to be defined and archived for future reference. *dtmslice* allows fast geometrical modelling inside a preloaded tomogram fraction. Visual tracking of three-dimensional structures is supported by *dpktomo.montage*, which simultaneously presents several slices in a region, or *dpktomo.concurrent view*, which combines several local orthogonal views around a given area.

### 2.5. Modelling   

**Figure 2:**
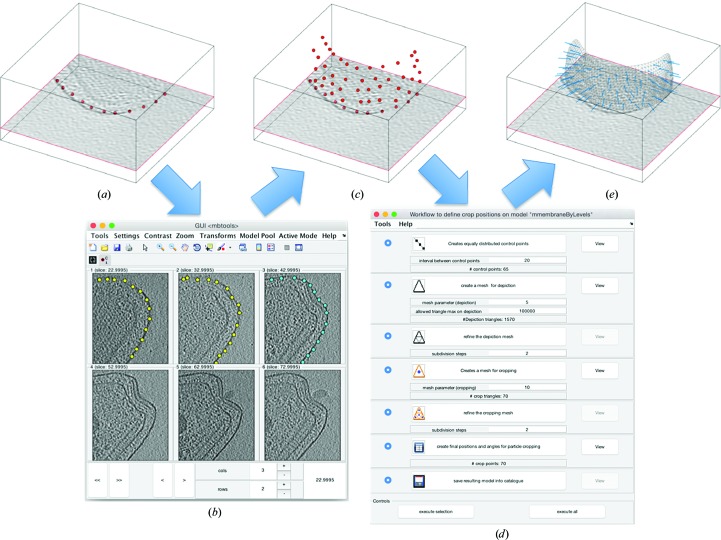
Model functionalities exemplified for membrane geometries. (*a*) shows a bacterial membrane where the user defines an area of interest in the tomogram browser and a group of seed points (in red). (*b*) is one of the user interfaces attached to the membrane model, offering a montage view of the area of interest. Each slice represent a *z*-cut of the tomogram for different, equispaced values of *z*. Seed points entered by the user on the first section are used to automatically compute points belonging to the membrane in the next section. The GUI allows the immediate evaluation and correction of the automatic detection. (*c*) The detected set of points. These are processed by the workflow GUI in (*d*) to parameterize the membrane as a smoothly triangulated surface, which is then used to define a regular distribution of points, each of which is provided with an initial orientation normal to the membrane, as shown in (*e*).

The annotation of tomograms runs through *models*. *Dynamo* includes a library of model types as described in Castaño-Díez *et al.* (2017 ▸) corresponding to different support geometries for particle cropping (vesicles, filaments, surfaces, pseudocrystals, isolated globular surfaces, isolated elongated surfaces *etc.*). On each of these geometries, the model fulfills two functions. Firstly, it drives the user to specific tools for volumetric data navigation that complement the generic tomogram browsers. As examples, filament-like models are supported by interactive depictions of sets of slices orthogonal to a hand-drawn path. Membrane-like models are connected with image-processing tools that allow the automated tracking of membrane points across consecutive sections of a tomogram, as depicted in Fig. 2 ▸. The second functionality is the design of *workflows* on the selected points. A model workflow provides a series of tools to stepwise build a mapping between selected points in a tomogram and a set of particles, describing their three-dimensional positions (and possibly orientations). In the membrane-like model, for instance, spline interpolation of the input points allows the creation of an equispaced set of control points, followed by a triangulation that smoothly characterizes the enclosed membrane in three dimensions and finally the definition of a set of particles regularly distributed on the membrane, each with an orientation given by the normal of the closest triangle. The parameters of such workflows (in this case the distance between control points, the mesh parameter that characterizes the triangulation and the distance between points) can be saved for systematic application to all selected models inside a catalogue.

**Figure 3:**
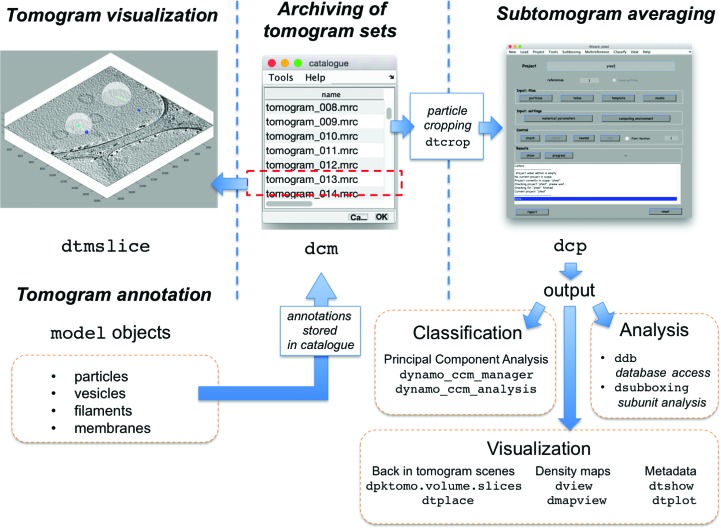
Functionality areas of *Dynamo* and associated commands. Catalogues (opened with the command dcm) take care of archiving lists of tomograms. Individual tomograms are visualized inside the volume browser dtmslice, with is connected to the model system and its geometric tools. The annotations created by models are stored in the catalogue, which can produce a data set of cropped subtomograms, a process driven by the command dtcrop. Alignment projects on the formed data set can be run through the GUI dcp. While the main result of an alignment project is the final average, in practice it is frequently necessary to closely inspect all of the results of an alignment process to further the analysis. To this end, *Dynamo* includes tools for PCA-based classification (dccm_analysis), visualization of averages (dview, dmapview) and metadata (dtshow) in the context of the original tomograms (dpktomo.volume.slices.Slice, dtplace) and database access to all elements generated by the project (ddb).

The interrelation among the main functionalities of the package is summarized in Fig. 3 ▸.

## 3. Software description   

### 3.1. *MATLAB*   

The codebase of *Dynamo* is mainly written in *MATLAB*. This design choice was taken in order to ensure fast and uncomplicated code production. This decision equally benefits the developers and users: historically, from the first practitioners the technique of subtomogram averaging required a significant amount of scripting, prototyping and testing, thus making *MATLAB* a natural choice.

The flexibility of *MATLAB* comes at the cost of slow performance when executing explicit loops on sets of pixels. *Dynamo* circumvents this limitation by the use of MEX files, which are pieces of code written in C++ whose compilation produces an executable viewed by *MATLAB* as a regular function. Therefore, variables defined in the workspace of *MATLAB* can be directly accessed by the MEX executables (*i.e.* without copying or transfer through disk) and processed by fast C++ code, with the generated results being visible from the *MATLAB* memory space. This approach is applied to accelerate operations that involve massive loops on pixels in a tomogram, such as three-dimensional rotations and I/O operations.

In addition to the MEX files explicitly written in *Dynamo*, *MATLAB* itself includes an extensive library of mathematical operations that have been *vectorized*, *i.e.* they are delivered as built-in precompiled functions and do not suffer any penalty derived from the execution of internal loops. Matrix-vector multiplications and multidimensional Fourier transform belong to this category.

MEX files and code vectorization ensure that the use of *MATLAB* does not introduce any slowdown in the *numerical* performance of *Dynamo* while running mathematical computations during alignment or classification projects. *Dynamo* is also structured to optimize its *graphical* performance (responsiveness of GUIs, latency while browsing tomograms) inside *MATLAB*. *MATLAB* versions subsequent to R2014b (*i.e.* released after 2015) integrate a major re­modelling of the *MATLAB* core that boosts the performance of the object-oriented programming (OOP) system and the graphics engine internally used to harness the application of OpenGL for the handling of graphical objects. *Dynamo* automatically inherits the performance increases that come with each semiannual *MATLAB* release. The fast OOP system of *MATLAB* ensures that the code maintainability in *Dynamo* does not come at the cost of reduced performance of the visual interfaces. The current graphics engine (R2016b) allows on-screen refreshing of matrices of 1000 × 1000 pixels in size at a rate of 36 fps on a MacBook laptop (2.9 GHz Intel Core i5, 16 Gb). This is sufficient to allow a smooth visual transition when browsing slices inside a binned tomogram.

### 3.2. Distribution to third parties   

*MATLAB* is commercial software. Users that are not interested in purchasing a *MATLAB* license can still install and use *Dynamo* outside the *MATLAB* environment. The standard *Dynamo* distribution contains a precompiled version of the package that includes all required libraries, known as MATLAB Compiler Runtime (MCR), which is distributed free of charge by MathWorks and deployed automatically in the user’s system during the installation of *Dynamo*. This *standalone* version provides all of the regular *Dynamo* functionalities: GUIs and commands for project creation, editing and execution are available, as well as the whole suite of tools for tomogram visualization and annotation provided by the *Catalogue* system.

The standalone version of *Dynamo* can be used in two ways: interactively by opening a *Dynamo* console, which allows the entering of commands and access to a workspace much like in a regular *MATLAB* session, or directly through the operative system. The syntax $ dynamo <dynamo command> orders the Linux shell to operate a single *Dynamo* command without opening a *Dynamo* session. This way of invoking *Dynamo* commands outside the interactive console incurs a time penalty: each time a command is invoked this way, the MCR libraries need to be initialized. Depending on the hardware and on the system configuration, this may cause a delay varying between fractions of seconds and 1 min. Additionally, commands invoked separately will belong to different variable workspaces, and will be able to interchange information only through the hard disk. For this reason, integration of *Dynamo* into third-party software is better accomplished through the syntax $ dynamo <command file>, where <command file> stands for a text file that contains several *Dynamo* commands, one per line, and may include programming tools as loops or conditionals. With this syntax, the initialization of the MCR libraries occurs only once and all the commands included in <command file> will also share the same memory workspace.

### 3.3. Generic *MATLAB* library   

In addition to streamlined workflows, *Dynamo* includes several specific modules for general tomography tasks, including alignment, annotation and reconstruction of tilt series, both for command-line and interfaced use. Expansions recently integrated in *Dynamo* rely on mbtools, which is a general-purpose *MATLAB* library originally conceived to support the development of *Dynamo*. It implements several development utilities that compensate for the lack of native tools in *MATLAB* for the production of large-scale code such as refactoring and visual editors (IDEs) for object-oriented programming. Its main modules deal with the automated creation of GUIs, the creation of workflows and flexible visualization and manipulation of generic streams of data. In the context of *Dynamo*, these abstract classes translate into unified browsers for navigating slices in a tomogram, particles in a set of extracted subtomograms, individual tomograms inside a catalogue or micrographs in a tilt series. As the mbtools are designed to avoid any *Dynamo*-specific dependencies, they have been used in other contexts, such as visualization and image tracking of light-microscopy data.

### 3.4. Parallelization   

The subtomogram-averaging pipeline in *Dynamo* handles several levels of parallelization: *fine-grain parallelization* concerns the parallel processing of a single task, while *coarse-grain parallelization* distributes the processing of different data particles among several devices operating in parallel. Fine-grain parallelization is performed by the multiprocessors of a GPU simultaneously computing each of the tasks required by the alignment of a single subtomogram.

Coarse-grain parallelization can be applied to the many processes in *Dynamo* that are executed through loops where the same operation is performed on different data units in an independent or weakly coupled way, such as the alignment of particles, the averaging of aligned particles and, in principal component analysis (PCA) classification tasks, the computation of elements in a cross-correlation matrix by independent blocks.

### 3.5. MATLAB Parallel Toolbox   

The MATLAB Parallel Toolbox offers a convenient way to parallelize pieces of code. A loop operated with the commands parfor/end instead of the regular for/end will execute the loop content (assuming no dependence) in a parallel fashion among a predefined set of processors. During the alignment step in a *Dynamo* project, the work unit of the loop content is the alignment of just one particle,
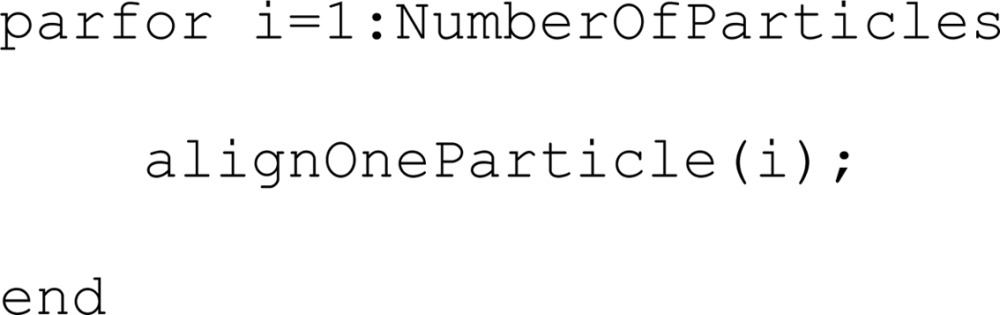


where the index *i* runs over all of the particle indices, with an unpredictable order of execution. Particles are presented as a *task queue* to the *thread pool* handled by the *MATLAB* parallel engine. Whenever a processor finishes the alignment of a particle, the pool immediately assigns to it a new, as yet unaligned particle. This approach inherently ensures an optimal workload balance among the cores during runtime, keeping all of them busy at any given time. This first-come-first-served scheme is preferred over the deterministic assignation of a predefined, equally sized set of particles to each core, as this may incur idle processors if the actual CPU time required for each batch is different and is not determinable before runtime (depending on the optimization method, some particles may need to scan more angles than others; also, some cores may be more powerful than others, or some of them may be slowed down by other operations running simultaneously in the system). This functionality is included in the standalone version of *Dynamo*, so that a Parallel Toolbox license is not necessary to run multicore projects in single nodes.

### 3.6. Cluster computing   

Using *Dynamo* on several nodes (as in a CPU cluster) is the only scenario where the provided standalone version is not a simple compiled version of the *MATLAB* code. Running *MATLAB* on a cluster requires an additional MathWorks product: the Distributed Computing Server (MDCS). If such a license is available, *Dynamo* will run smoothly without any further installation. In the case that an MDCS license is not available for the cluster, the package can still be installed as a stand­alone. Unfortunately, the MDCS is not compilable. In other words, there is no compilable tool that allows the creation of a single *MATLAB* instance that controls computing threads in different nodes in the same memory workspace. Instead, the standalone version for clustering operates by spawning different MPI tasks, each one a *de facto* independent *MATLAB* process (all of them running against the same MCR library) and with a different memory space. Different instances of *MATLAB* can communicate only through file I/O, precluding the creation of a single, unified thread pool. For this reason, the basic implementation of *Dynamo* for clusters just assigns a set of particles to each core before runtime. This procedure is unable to balance the workload in clusters with heterogeneous resources, and is also unable to handle single cores failing to complete their task because of hardware or system failures (both of which are rather frequent events in large-scale computing installations). From version 1.2.*x*, the MPI parallelization works on a first-come-first-served basis. In opposition to the *MATLAB* native task pooling used for the single-node case, communication between threads residing in different nodes occurs through *file semaphores*. A compiled *MATLAB* instance will access a new particle only if a semaphore (defined on disk and visible to all of the instances) is open. *Dynamo* implements the semaphore in two variants: if the user knows the mkdir command to be atomic in the cluster file system (a condition not necessarily met in NFS mounted file systems), *Dynamo* will run out of the box in the cluster. Otherwise, a second semaphoring system can be used. This fallback system is based on the Linux command lockfile, which is NFS-resistant. Unfortunately, it is not part of most standard Linux distributions, and will require installation by the user previous to the use of *Dynamo.*

### 3.7. Parallelism in generic functions   

Several *Dynamo* commands that can be invoked from the command line offer the option to be executed in parallel using the MATLAB Parallel Toolbox. Some commands relate directly to the parallelization of operations on groups of particles, such as dtcrop, which is used to extract subtomograms from (sets of) tomograms, daverage or ddcheck, but the option is generally available in any command that implements a parallelizable operation; for instance, example scripts that perform the same lengthy processes on each model on a group (for instance, the computation of smooth triangulation grids that describe membranes) or operations on full tomograms (filtering, binning), where the volumetric data are automatically distributed in chunks that are sent to different processors.

Users can check whether a particular command allows parallel execution through checking the documentation of the function (help or doc). By syntax convention, the inclusion of the flag -mw (or -matlab_workers) followed by the number of physical cores sends the order to the thread pool. When the -mw flag is invoked through a *MATLAB* session and a Parallel Toolbox license is not available, *Dynamo* will issue a warning message and run in single-core mode. If the command is invoked through a standalone session, no license is needed.

### 3.8. GPU computing   

Graphic processing units (GPUs) are special-purpose processors that were originally developed for calculations required in three-dimensional graphic rendering and other display functions. Since the late 2000s, they have been increasingly used for general-purpose calculations in scientific computing. Several packages for electron microscopy include support for GPUs in tasks such as movie alignment (Li *et al.*, 2013 ▸), tilt-series alignment (Castaño-Díez *et al.*, 2010 ▸), tomographic reconstructions (Castaño-Díez *et al.*, 2007 ▸; Vázquez *et al.*, 2010 ▸), subtomogram averaging (Galaz-Montoya *et al.*, 2015 ▸), single-particle analysis (Kimanius *et al.*, 2016 ▸), particle picking and CTF determination (Zhang, 2016 ▸).

The speedup delivered by a GPU in relation to a CPU is highly dependent on the algorithm. Despite the high memory bandwidth of modern GPU devices (480 Gb s^−1^ in K80 or 336 Gb s^−1^ in Titan X), they demonstrate their full potential only in *compute-intensive* applications, *i.e.* algorithms where the data-transfer requirements are significantly below the actual computing time. Subtomogram averaging is thus a paramount example of suitability for GPU computations: after loading a particle, a template and a mask into the GPU, the device proceeds to execute a large loop of the scanning angles, each step comprising several volumetric computations as rotations and Fourier transforms.

### 3.9. GPU in *Dynamo*   

*Dynamo* includes two GPU implementations of the alignment procedure. The *compact* version is fully written in CUDA/C++. During the runtime of the project, the *MATLAB* codebase of *Dynamo* simply operates a system call that invokes a single GPU-oriented executable which aligns all of the particles in the data set. This executable handles the whole logistics of the particle alignment, including I/O actions, loop execution and mathematical operations, duplicating the functionality of the equivalent *MATLAB* code that would be used in the CPU. If several GPU devices are available, each one will be governed by a different CPU thread and will align an equal number of particles. Particle assignment is decided in the CPU before runtime of the GPU code, and communication between the *Dynamo* project and the spawned GPU computations occur exclusively by file exchange through the hard disk.

From *Dynamo* v.1.2.*x*, the software also includes a *modular* implementation that directly embeds GPU kernels into the main *MATLAB* code. This approach makes use of the newly introduced gpuArray type. This data-storage type allows one to keep and manipulate objects in the memory of a GPU device directly from the *MATLAB* workspace. Many of the functionalities needed for alignment are already included in *MATLAB* as optimized and GPU-enabled built-in functions (Fourier transforms and linear algebra); the missing functionalities are provided by *Dynamo* as precompiled kernel objects. These kernel objects parallelize in the GPU the function of MEX files in the CPU: they are written in C++/CUDA and their executable can share memory with variables residing in the *MATLAB* workspace, in particular with gpuArray variables physically located in the GPU device.

This software design implies an enormous flexibility towards future developments, ensuring that *MATLAB* code prototypes devised for the CPU can be immediately ported for GPUs. For users, this modularity allows the effortless use of GPU-enabled *Dynamo* functions (as three-dimensional rotation or backprojection) in their own *MATLAB* code. Further, the CUDA kernels provided in *Dynamo* are not *MATLAB*-specific, and documentation is provided so that users can compile them into their own C++ or Python applications.

Additionally, the smooth integration of GPU/CPU code provided by the modular version allows one to launch mixed GPU/CPU pools through the *MATLAB* Parallel Toolbox. This way, in a machine with 64 cores and a GPU, *Dynamo* can reserve one of the cores to control a GPU process to align particles extracted from a task pool simultaneously alongside the remaining 63 CPU cores, optimizing the use of all available processing resources in a single node at any given time.

### 3.10. Distribution of GPU code   

When used in *MATLAB*, the GPU modular version needs a particular version of CUDA (compatible with the *MATLAB* version run by the user) and a license for the MATLAB Parallel Computing Toolbox. If this is not the case, the compact GPU version can be executed (after compilation by the user against the local CUDA libraries) both inside *MATLAB* or through the standalone version.

### 3.11. GPU devices   

The NVIDIA company delivers GPUs that are specifically conceived for scientific computing. These devices include an error-correcting code (ECC) mechanism that protects the memory from random corruption events. While the use of ECC memory is necessary for numerically sensitive procedures, its use introduces a considerable fabrication cost. The bulk of the computations performed in subtomogram averaging is directed towards the calculation of single scalars that define the similarity of pairs of volumes by cross-correlation. Such procedures are unlikely to be affected by rare, spatially isolated bit flips inside volumetric data, making the use of less expensive, non-ECC devices a possibility.

### 3.12. Performance comparison   

We have tested the speedup attained by different devices compared with a single CPU core during the execution of a single three-dimensional fast Fourier transform (FFT) in single precision. The time estimation was performed in *MATLAB* by repeated computation of an fftn command on the same array of data inside a for/end loop and estimating the cost of a single operation as the total time divided by the number of loop steps. This command uses the CuFFT (Jodra *et al.*, 2015 ▸) libraries when its argument is of type gpuArray (*i.e.* is located in the GPU), and the FFTW libraries (Frigo & Johnson, 1998 ▸) otherwise, thus delivering state-of-the-art performance for both the GPU and CPU.

The loop was evaluated several times with increasing lengths until consistent results were achieved. This comparison procedure ignores the memory-transfer overhead. This approach is realistic, as subtomogram-averaging computations will typically launch large amounts of fftn computations on pieces of data already residing in the GPU memory.

As a CPU reference, we used an Intel Core i5-5287U at 2.9 GHz in a MacBook Pro. This provides a fair baseline for a performance comparison with GPU devices, as this CPU was found to outperform the core-by-core processors frequently used in large multicore machines (the Xeon E5-2697 v3 at 2.60 GHz and the Xeon E5-2630 v3 at 2.40 GHz were tested).

*MATLAB* automatically multithreads FFT computations. In order to determine an accurate comparison of a single core with a GPU, the CPU fftn commands were run in parallel and the total time was divided by the number of physical cores.

The tested GPU cards have different architectures and price ranges, as reported in Table 1 ▸.

The K620 is a classical graphical card, intended for display control and with limited computing resources. Titan X and GTX 1080 are non-ECC devices from the newest generation (Pascal); note that there is a device also called Titan X from a previous generation (Maxwell). A previous architecture, Kepler, is represented by the high-end K20m and K80, furnished with ECC memory. The device commonly known as K80 is in fact a dual card consisting of two GK210 units; the results reported here were initially computed on such a single unit and extrapolated for the performance of two. For all cards, prices in US$ were obtained as of December 2016 and are merely orientative. These prices were used for the columns marked as ‘speedup per $1000’ in Table 2 ▸.

Note that the reported speedups do not directly translate into the speedups attained by *Dynamo* in the runtime of an actual project: Table 2 ▸ is merely intended as an orientational guide for comparison among GPU devices and an estimation of the expected value. If the user accepts the lack of ECC memory, the Pascal-based cards presented here are the best choice, with Titan X providing the best performance and GTX 1080 providing the best performance in relation to its price. Interestingly, even a commodity GPU such as the GTX K620, which was devised mainly for on-screen image rendering, is able to deliver a noticeable (and inexpensive) speedup over a single core.

### 3.13. Cloud computing   

A research group that considers embarking on the use of GPU devices might face some initial difficulties. While purchasing and installing a single card in a previously available workstation may be inexpensive and straightforward, the setup of a powerful, dedicated GPU server requires a larger ‘activation energy’. In addition to the price of the GPU devices and a rackmount server to harness them, the available hardware infrastructure (data centre) needs to be prepared to host the GPU units in terms of energy supply and ventilation. Thus, a research group that needs only occasional access to GPU resources might become discouraged by the initial investment of time and effort.

An obvious alternative is the use of externally maintained servers. GPUs are frequently offered by the centralized clusters maintained by hosting research institutions and by other dedicated facilities as national supercomputing centres. Even when such resources are not available, not sufficient or not convenient, users can resort to commercial solutions.

### 3.14. Computing on Amazon EC2   

The Amazon company provides access to virtualized computing environments through its Amazon Web Services (AWS). Using the Elastic Compute Cloud (EC2), users with an AWS account can request access to the computing resources needed for a given numerical experiment, paying only for the computation time actually used, and enormously simplifying the access and use of GPU machines: initial and overhead costs disappear, no further hardware maintenance and upgrade is required, system administration is run by Amazon, and NVIDIA provides correctly configured virtual environments. Furthermore, all resources (computing power, memory and storage) can be flexibly scaled to the needs of the current project.

The use of single nodes of the EC2 cloud runs through highly configurable virtual servers called *instances*. Launching an instance requires the selection of a software setup and a physical hardware configuration (storage requirements can be flexibly determined before launch or adjusted during runtime). The software setup defines the tools and the system environment seen by the instance at launch time. Templates for software setup are called AMIs (Amazon Machine Images). Amazon provides a series of basic AMIs, complemented by a large library of third-party contributions (AWS Marketplace), which are both commercial and free. The preconfigured environment provided by the chosen AMI will run on a hardware defined by the selected *instance type*. Amazon provides a series of fixed hardware configurations, with different number of CPUS, GPUS and RAM resources. Once the instance is launched and running, it can be accessed by a regular ssh command and used in the same way as any other remotely accessed server terminal.

Storage space is purchased independently as one of the configurable options of the instance (prices vary per region and are typically in the range 0.02–0.03 US$ per Gb per month as of December 2016). Regular data transfer incurs no cost, while accelerated data transfer is charged at 0.04–0.08 US$ per Gb.

### 3.15. Preconfigured *Dynamo* environment   

*Dynamo* maintains a ready-to-use, publically available AMI. This AMI contains an installation of *CUDA* 7.5 with suitable drivers and a full installation of the *Dynamo* stand­alone, including all necessary libraries. An EC2 user that launches an instance of the *Dynamo* AMI just needs to ssh into the running instance and type the command dynamo in the terminal to start working on a *Dynamo* project.

AWS provides several types of GPU-enabled servers based on the K80 card. The most powerful one is the p2.16xlarge type. Each instance of this type provides eight K80 cards (and thus 16 GPU units, as the K80 is a dual card) complemented with 32 physical CPU cores (64 logical cores) and 732 GB of memory. *Dynamo* will use these additional CPU cores to parallelize the averaging step and in parallel with the GPUs during the alignment step.

While the *Dynamo* AMI is provided for free, users pay for the time consumed by their instance. As of December 2016, the maximum cost per hour of a p2.16xlarge instance is ∼14 US$. This fixed price corresponds to on-demand request of computing time to immediately start an interactive session. Running instances with flexible timing schemes (*On Spot instances*) can decrease the cost by 90% from the on-demand price, depending on the punctual supply and demand situation of the requested instance type.

The *Dynamo* AMI can be used on CPU-only instances. This, however, is not cost-beneficial: depending on other configuration factors (mainly RAM and network performance) the cost per CPU processor and hour is about 0.22 US$ (using the best instance type, based on an Intel Xeon E7 8880v3 at 2.3 GHz s^−1^; December 2016 prices). The price per K80-based GPU processor and hour is four times greater: 0.88 US$. As the speedup factor provided by a single GPU in *Dynamo* typically ranges between 15× and 40× (depending on the CPU and the problem size), computing time in GPU instances is roughly four to ten times cheaper.

Notice that the GPU capabilities currently offered by Amazon are based on the K80 architecture, which does not deliver an optimal performance:cost ratio. A potential future expansion of the EC2 based on Pascal-type GPUs would be likely to cause a significant cost reduction.

## 4. Conclusions   

The *Dynamo* package offers a flexible software infrastructure for tomography-related projects, embracing most of the possibly required processing steps inside an integrated environment. Its internal structure exploits the simplicity of the *MATLAB* programing language in several aspects, in particular its parallelization and GPU-enabling engines, the application of which inside *Dynamo* 1.2.*x* has been described and documented to facilitate their use as modular components in other applications. *Dynamo* aims at helping cryo-electron tomography practitioners to adopt the GPU technology as a standard computing device, especially for subtomogram averaging. Advanced users or developers are provided with ready-to-use tools that greatly simplify the production of efficient GPU code. New users have the option of simply running *Dynamo* GPU projects in the cloud on a pay-as-you-go basis, avoiding costly initial purchases and the complex software and hardware setup.

## Figures and Tables

**Table 1:** Features of compared GPU models

GPU model	CUDA cores	Clock speed (MHz)	Price estimate ($)
Quadro K620	384	1059	190
GTX 1080	2560	1670	700
Titan X Pascal	3072	1000	1700
K20m	2496	706	2200
K80 (dual)	2 × 2496	562	4400

**Table 2:** Speedup attained by the tested GPUs for three-dimensional (3D) FFT computations at different cube sizes

		CPU									
		K620		GTX 1080		Titan X		K20m		K80	
Cube size	Time per 3D FFT (ms)	Speedup	Speedup per $1000	Speedup	Speedup per $1000	Speedup	Speedup per 1000$	Speedup	Speedup per $1000	Speedup	Speedup per $1000
32	5.8	35×	176	45×	64	49×	27	38×	17	100×	23
64	6.2	16×	78	45×	65	50×	28	39×	18	102×	23
128	68.3	14×	71	131×	188	179×	100	78×	36	198×	43
256	566.0	13×	67	178×	254	261×	145	100×	45	236×	54
512	6719.8	—	—	137×	296	202×	112	73×	33	178×	40
